# A Ray of Hope: Gamma Radiation for Microplastic Remediation

**DOI:** 10.1002/gch2.202500117

**Published:** 2025-05-07

**Authors:** Dhanalakshmi Vadivel, Claudio Casella, Adriana Laca, Mario Díaz, Daniele Dondi

**Affiliations:** ^1^ Department of Chemistry University of Pavia Viale Taramelli 12 Pavia 27100 Italy; ^2^ Department of Chemical and Environmental Engineering University of Oviedo C/ Julián Clavería s/n Oviedo 33006 Spain

**Keywords:** dosage and ^60^Co, gamma irradiation, microplastics (MPs), sewage sludge

## Abstract

Urban wastewater treatment plants (WWTPs) generate sewage sludge, which retains ≈95% of the microplastics (MPs) processed in wastewater. The literature describes sewage sludges with MP concentrations ranging between 400 and 170,000 particles kg^−1^ (dry weight) and when this sludge is applied to land, MPs spread into the environment. As a possible treatment for sewage sludge, this study aims to evaluate the effect of gamma‐rays on the degradation of sludge MPs. The MPs utilized in the experiments are obtained from secondary sewage sludge provided by a municipal treatment facility and they are physic‐chemically characterized (size, shape, composition). MPs are exposed to gamma radiation (γ‐irradiation) at different time intervals and total doses to study the response process in sewage sludge. In particular, they are treated with γ‐irradiation using cobalt‐60 (^60^Co) at doses ranging from 0 to 116 kGy. In this study, MP degradation can be achieved with a maximum degradation percentage of almost 70%. Concerning the specific degradation, research results show that both MP forms exhibit the same 35% degradation rate. This study not only advances the knowledge of how γ‐irradiation affects MPs, but it also opens a new approach to fight against the global problem of environmental MPs.

## Graphical abstract

Gamma irradiation is a promising method for degrading microplastics in sewage sludge. Cobalt‐60 doses up to 116 kGy are used, achieving over two‐thirds degradation at the highest level. With further optimization, gamma irradiation could improve wastewater treatment sustainability by minimizing microplastic presence in biosolids and protecting ecosystems.

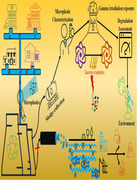

## 1 Introduction

A global assessment projected that from 1950 to 2015, ≈4.9 billion metric tons of plastic garbage entered landfills or the natural environment, with projections suggesting it could reach ≈12 billion tons by 2050. If these trends persist, plastic pollution will constitute a global catastrophe.^[^ ^1^ ^]^ In 2023, 413.8 million metric tons of plastics were produced worldwide. The European value was 54 metric tons.^[^ ^2^ ^]^ MPs are small particles of plastic, usually less than 5 mm^[^ ^3^, ^4^ ^]^ which are classified into two most common types: Primary and Secondary. Primary MPs include the microbeads used in cosmetics, personal care products as well as industrial abrasives; they also involve plastic pellets used during the production process.^[^ ^5^, ^6^ ^]^ Secondary MPs are the result of the degradation of bigger plastic objects, including water bottles, plastic bags, and synthetic fabrics.^[^ ^7^ ^]^ Over time, these larger things gradually disintegrate into smaller particles due to sunlight, wind, and wave action.

MPs are ubiquitous in the environment, ranging from the oceans and rivers to the soil and even in the air.^[^ ^8^ ^]^ They pose substantial hazards to both marine and terrestrial ecosystems. This contamination may cause in living organisms physical damage, blockages, and even exposure to toxic chemicals that are adsorbed onto the plastic.^[^ ^9^ ^]^ MPs can enter into the food chain^[^ ^10^, ^11^ ^]^ when consumed by organisms and move up to humans. According to the EU Commission in 2017, MPs pose a particular concern because of the severe adverse effects they are causing on marine and freshwater environments, aquatic life, biodiversity, and possibly human health. Their tiny size enables their easy uptake and bioaccumulation by organisms; besides, the complex mixture of chemicals they consist of might cause toxic effects.^[^ ^12^ ^]^ It has been reported that MPs could cause toxicity to the digestive system, respiratory system, cardiovascular system, nervous system, reproductive system, immune system, and endocrine system.^[^ ^13^ ^]^ Long‐term MP intake might alter our human chromosomes and result in obesity, infertility, and even cancer.^[^ ^14^ ^]^ Moreover, the estimated annual consumption of MPs is estimated to fluctuate from 39,000 to 52,000 particles per person yearly, depending on both sex and age.^[^ ^15^ ^]^

Polyethylene terephthalate (PET), polyvinyl chloride (PVC), polystyrene (PS), and polypropylene (PP) are the most common types of plastic found in environmental sludges.^[^ ^16^ ^]^ Undeviatingly, due to their large surface area, and high surface hydrophobicity, MPs easily adsorb contaminants like microorganisms, heavy metals, and organic pollutants.^[^ ^13^ ^]^ Heavy metals, chemical additives, pesticides, organic pollutants, and persistent, bioaccumulative, and toxic compounds are among the various harmful substances found in plastics.^[^ ^17^ ^]^ In addition, dyes, which are a chemical constituent of colored plastics, are designated as priority pollutants by the United States Environmental Protection Agency (USEPA).^[^ ^18^ ^]^ The issue of eliminating MPs from aquatic bodies is compounded.

MPs are a problem because they contaminate sludge and sediments and they make it harder to treat wastewater and sewage sludges just using traditional methods like coagulation, air flotation, and membrane filtration.^[^ ^19^ ^]^ The catalytic decomposition of bulk plastic trash has been considered and discussed in depth by a number of articles.^[^ ^20^ ^]^ Recently, Chen et al. discussed the recovery of waste plastics for recycling using different catalytic approaches, such as thermocatalysis, electrocatalysis, biocatalysis, and photocatalysis.^[^ ^21^ ^]^ Farahbakhsh et al. recently studied the removal of MPs with dyes by using ultra‐filtration techniques in a primary stage.^[^ ^22^ ^]^

Considering the substantial possession of polymer^[^ ^23^ ^]^ based properties of MPs, it is pivotal to understand their origins, distribution, migration, fate, effects, and potential solutions. Advanced oxidation processes (AOPs) are currently the predominant technique for remediating MPs^[^ ^24^ ^]^; nonetheless, the depletion in MP degradation efficacy following several testing cycles, especially in photocatalytic oxidation utilizing catalysts, demands careful consideration. In such a respect, it becomes very relevant to select appropriate degradation processes for MP treatment, depending on the type of MPs. While these technologies are in their infancy at present, further research and development is in urge to deal with MP degradation which plays an important role in sustainable development in reducing pollution from plastic waste.^[^ ^25^ ^]^

The issue of contaminant contamination in sewage sludge, which is hazardous to human health, can be resolved with radiation technology. Additionally, sewage sludge can be exposed to high‐energy radiation from radioactive sources like ^60^Co to successfully eradicate any pathogens present. ^60^Co can inactivate viruses effectively, cleanly, and with a high degree of reliability. The radiation effect is guaranteed under a thick layer of sludge because gamma‐rays are very permeable to both water and sludge.^[^ ^24^ ^]^ According to Wang et al.,^[^ ^26^ ^]^ the half‐value thickness of Cesio‐137 (^137^Cs) is 24 cm in water, whereas the half‐value thickness of ^60^Co (1.3 MeV) gamma‐rays is ≈28 cm in water and at least 25 cm in regular liquid sludge. Depending on the thickness and volume of the product, the γ‐irradiation period can range from minutes to hours.^[^ ^24^ ^]^ The development of integrated sludge management techniques would help address national public health and sanitary engineering issues related to the safe disposal of sewage sludge, as well as recover the environmental value of sludge for use as safe fertilizers in agricultural practices.^[^ ^27^ ^]^ The first gamma irradiator for disinfecting sewage sludge was set up at the Geiselbullach treatment plant in Germany. The radiation sources had a cylindrical shape, measuring 300 mm in length and 30 mm in diameter. There were 30 ^60^Co platelets in each. An additional source was added every two to three years. About 5.6 m^3^ of sludge was treated by the irradiator in batch mode, with an absorbed dose of 3 kGy.^[^ ^26a,b^ ^]^ In 1979, Sandia National Laboratories in Albuquerque, USA, constructed and operated a gamma irradiation pilot plant to treat dewatered and composted sludge that was also combined with other solid waste. ^137^Cs was the radiation source. With a necessary dosage of 10 kGy, the intended capacity was 8 tons of dry sludge (≈50% dry solids) each day. However, for unspecified reasons, the plant's operations were halted (Lessel T., 1997). Compared to traditional processing methods, sludge irradiation has special benefits. Sludge irradiation is probably going to become standard procedure as accelerators get comparatively cheaper and waste becomes more of a concern. Examples of advantages include the fact that no heat energy is needed, that the equipment and process are straightforward, dependable, and easy to manage, that no chemicals are applied, that radiation efficiently inactivates germs in the sludge, etc.^[^ ^27^, ^28^ ^]^ In a process known as“radiolysis,” gamma‐rays physically break down or degrade MPs into simpler chemicals through interactions between organic molecules and the energy of ionizing radiation. Gamma‐rays generate free radicals, which are extremely reactive chemical entities that target the polymer chains of MPs, breaking them up into smaller pieces or changing their chemical makeup to help break them down in the environment.

Through a comprehensive grasp of the difficulties relevant to sustainable development, we propose a novel degrading technique to address MP depletion. In this challenge, we are utilizing several types of MPs, specifically fibers, and fragments, extracted from sewage sludges. The chemical composition of the MPs was determined by using µ‐FTIR and the ranges of sizes and shapes were analysed by stereomicroscopy. In this study, all MP samples were subjected to γ‐irradiation in varying doses up to 116 kGy to assess the effects of irradiation and the necessary dosage. This study aimed to investigate the capacity of MPs in sludge to degrade utilizing gamma‐rays as an alternative technique for removing or reducing these pollutants, which pose a major risk to human health and the environment. To the best of our knowledge, no prior study has measured the extent to which gamma irradiation degrades MPs (unless certain characteristics are taken into account, such as the work by Mikac et al.).^[^ ^28^ ^]^ MP samples taken from secondary sludge were subjected to gamma irradiation in an aqueous solution as part of this investigation. Spectrophotometry and micro FTIR spectroscopy were used to identify and measure the resultant MP particles.

## 2 Results and Discussion

**Figure 1:**
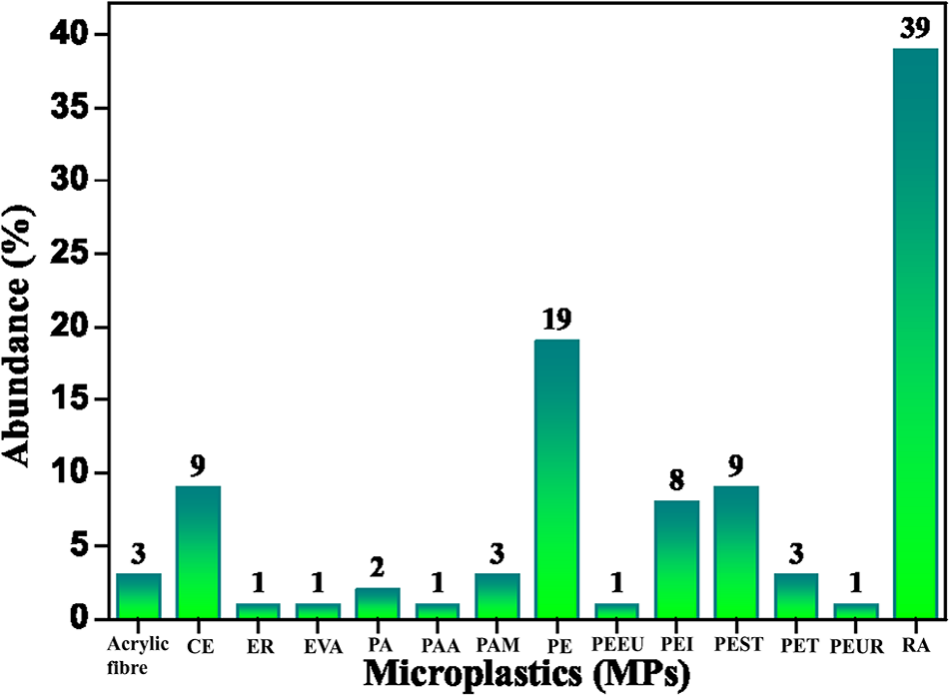
The chemical composition of MPs was analyzed in secondary sewage sludges.

Examining the chemical composition of MPs in sewage sludge is crucial for several reasons. First, understanding the kinds of polymers that comprise MPs aids in evaluating possible risks to the environment and human health. Second, knowing the chemical makeup of MPs could increase the efficacy of procedures to remove them from sludge. Furthermore, MPs' chemical makeup particularly their capacity to absorb and transport pollutants will determine how they interact with the environment. To enable policymakers^[^ ^4^ ^]^ to create efficient mitigation plans, accurate analysis guarantees adherence to environmental norms and laws. The chemical composition of the MP collected in the secondary sewage sludge is shown in **Figure** **1**. The maximum abundance is seen by rayon (RA) (39%). Because these fibers are not biodegradable, they are released during laundry and stay in the sludge. Studies show that polyethylene (PE) particles make up a significant portion of the microplastics found in sewage sludge, frequently in combination with other plastics such as polyethylene terephthalate (PET) and polypropylene (PP). Figure 1 depicts an abundance of roughly 19% for PE and 3% for PET. PE (19%) is used in packaging, plastic bag fragments, synthetic garments (blends with polyester, PEST), and as a scrubbing agent. Cellulose (CE) shows an abundance of 9% in the analysis of sewage sludge because it is used as a thickening component in industrial additives (paints, adhesives, textiles), personal care products (creams, scrubs), foods, etc. PEST (9%) is found, as is CE. The primary source of PEST is textiles and clothing. PEST garments, which are commonly seen in sportswear and everyday clothing, release tiny fibers during the washing process, which are then eliminated with the wastewater. Additionally, it is utilized in many other products, such as plastic bottles, films, and packaging. Subsequently, polyethyleneimine (PEI) is found in wastewater loads at a rate of 8%. This is less frequent than other contaminants like PE or PEST, but it can still happen because of certain industrial uses and its resistance to biodegradation. Its primary uses include insulators and membrane components for liquid and gas filtration, as well as medical equipment (surgery tools and supplies).

The purpose of the research was to assess gamma radiation (𝛾‐irradiation) for the degradation of MPs from sewage sludge. Samples containing the two different prevailing shapes, i.e. fibers and fragments, were suspended in water, as explained in the Experimental Section, and subjected to 𝛾‐irradiation treatment. The percentages of MP elimination using gamma‐rays [AP1] were calculated for fibers and fragments, and are shown in **Tables** **1** and **2**. 𝛾‐radiation was more effective in degrading fragments than fibers for all the doses tested. MP fragments are generally more susceptible to γ‐irradiation due to their lower structural complexity and greater surface exposure. More frequent attacks by free radicals on the surface of the fragments can hasten their chemical and physical degradation. MP fragments are typically more susceptible to γ‐irradiation because of their increased exposed surface area and decreased structural intricacy. MP fibers may be more resistant to gamma radiation because of the longer and denser structures of MP fragments, which are present in synthetic textiles like nylon/polyamide (PA) and PEST. Furthermore, the threads could tangle or partially hide their internal surfaces, making it harder for free radicals to degrade them.

**Table 1:** Comparison of total percentage degradation of MPs with gamma‐rays (MP shape: fibers and fragments).

Gamma‐ray ray total dose [kGy]	% MP shape		% MP degradation		% MP Total degradation
	Fibers	Fragments	Fibers	Fragments	
50	69	31	6.7 ± 7.6	14.2 ± 8.4	9.7 ± 2.8
86	71	29	22.5 ± 9.8	47.7 ± 12.7	32.7 ± 3.2
116	65	35	62.9 ± 6.4	72.3 ± 3.1	66.2 ± 4.3

**Table 2:** Comparison of percentage degradation of MPs with gamma‐rays (MP size: fibers and fragments).

Gamma‐ray total dose [kGy]	MP size [µm] before irradiation		MP size [µm] after irradiation	
	Fibers	Fragments	Fibers	Fragments
50	786 ± 51	632 ± 89	699 ± 37	558 ± 96
86	858 ± 67	645 ± 152	571 ± 46	431 ± 106
116	452 ± 96	385 ± 31	295 ± 70	248 ± 20

**Figure 2:**
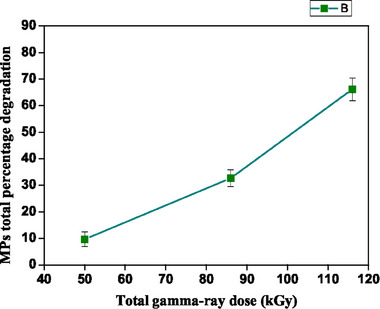
Deterioration of the total percentage of MPs shape for fiber and fragment.

**Figure 3:**
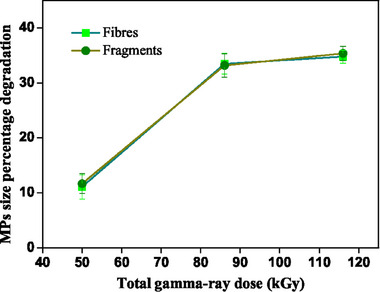
Percentage of MP size degradation for fiber and fragment.

This difference is less evident for the highest dose assayed since an important increase in the removal efficacy, from 22.5% to 69.2%, was observed for the fibers when the dose increased from 86 to 116 kGy. As can be seen in **Figure** **2**, the total removal efficacies were proportional to the irradiation doses. In **Figure** **3**, the total size percentage degradation of MPs is mentioned for both fibers and fragments, which shows ≈40 % degradation when the applied gamma‐rays are at the maximum of 116 kGy.

**Figure 4:**
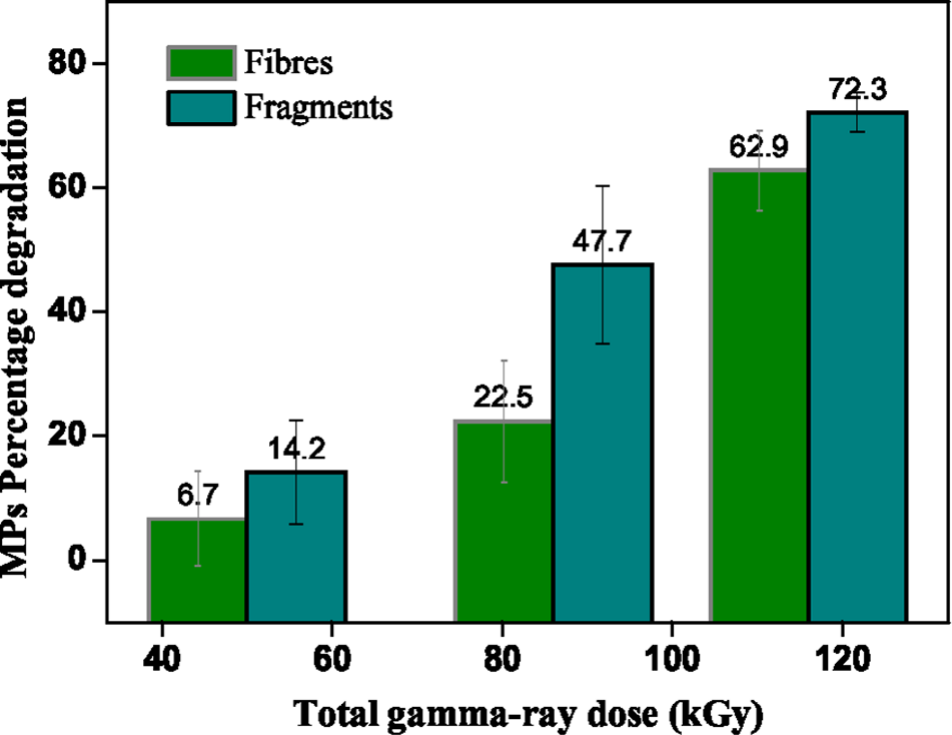
The rate of MPs fiber and fragment degradation as a percentage.

As MPs were quantified by counting, the efficacies were calculated considering just the elimination of MPs. However, before being eliminated MPs are likely to be gradually degraded, reducing their size. To confirm this hypothesis, the size of MPs was measured before and after being treated with gamma‐rays, and average values are shown in Table 1. As can be observed, both fibers and fragments reduced their average size for all doses, exhibiting similar behavior. This proves that, if this reduction in the size of the remaining MPs is considered, the degradation efficacies would be even higher than those shown in **Figure** **4**. About the irradiation dose employed, as expected, the reduction in size (and therefore the degradation) was higher for higher doses, in particular when the dose increased from 50 to 116 kGy.

In literature, Siwek et al. investigated the efficacy of electron beam treatment in the disintegration or removal of MPs from wastewater and sewage sludge in different doses. They are able to degrade above 70% of MPs during their study.^[^ ^29^ ^]^ Also, Mikac et al., the impact of 𝛾‐irradiation on PET and recycled PET. They are able to degrade a reasonable amount of the MPs.^[^ ^28^ ^]^ In the same way, we are able to degrade a reasonable amount of MPs size and shape degradation in the two different forms that are fragments and fibers. Certain polymers, like PE or PP, require larger doses than more “oxodegradable” plastics, indicating that the effectiveness of MP removal varies by polymer type. Furthermore, the absorbed dose and γ‐irradiation flow (irradiation must be homogenous to treat the samples efficiently) have an impact, as do the characteristics of the medium (pH, organic matter, oxygen content, and other chemicals might affect efficiency). Sewage sludge requires larger doses of γ‐irradiation (≥ 30 kGy) because of its increased density and organic content. We have chosen to employ ^60^Co in this work because of its high energy (1.17 and 1.33 MeV) and because it is the most commonly used gamma‐ray source in both research and manufacture. It can be effective for the degradation of MPs in liquids or complex mixtures due to its high level of penetration into the material, producing free radicals in aqueous conditions, which accelerate the chemical decomposition of polymers. In specialized facilities, the source is reliable and under control. Due to its high level of material penetration, it can be useful for the degradation of MPs in liquids or complicated mixtures. In aqueous settings, it produces free radicals, which hasten the chemical breakdown of polymers. The supply is controlled and dependable in specialized facilities.

**Figure 5:**
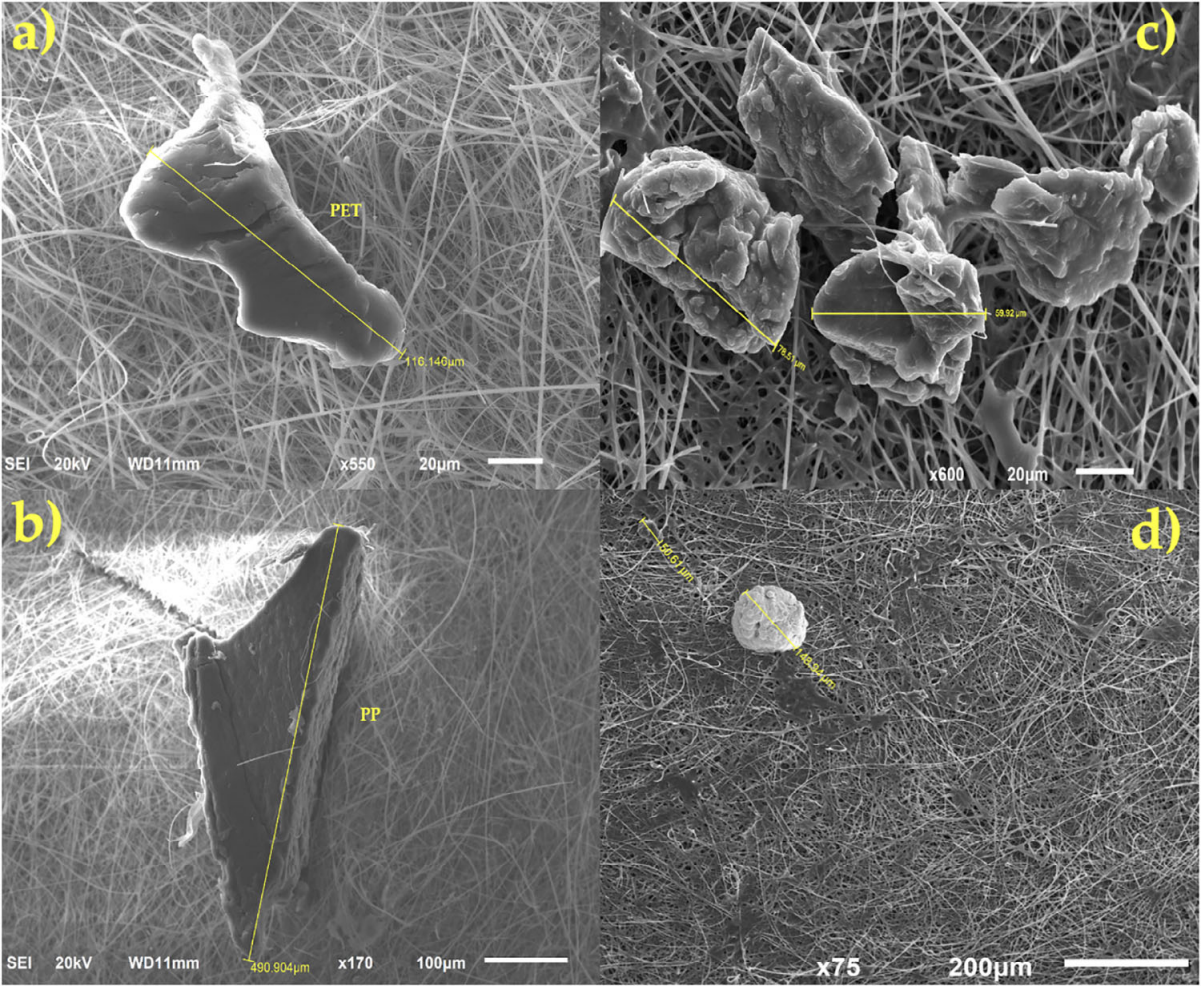
SEM images of the MPs: PET and PP fragments are observed in Figure 5a,b before irradiation. MPs fragments can be detected in Figure 5c following 50 kGy irradiation, and in Figure 5d after 116 kGy irradiation.

We compared the morphology of MPs pre‐ and post‐gamma irradiation using SEM analysis. (**Figure** **5**). When MP fibers and fragments are exposed to gamma irradiation, radiation‐induced degradation can lead to a variety of physical‐chemical alterations. Slight fragmentation and size reduction (scission effects; cutting of polymer chains) can be observed already at an intensity of 50 kGy. In MP fibers, this ends up in gradual weakening and the potential for microfibril formation. The radiation‐induced molecular reorganization may cause the fibers to become slightly bent and lose their alignment. MP fragments have tiny microcracks on the surface of the polymers, which makes it easier for the polymer to break up into smaller fragments later on. Additionally, if these fragments come into close contact, there may be uneven edges or even partial fusion (Figure 5c). Due to the slower rate of degradation of PE and PP compared to other polymer types (i.e., PA, PET, PS, etc.), these impacts are less noticeable in these cases. In Figure 5d, when exposed to 116 kGy, MPs fracture and show a significant size decrease. Color alteration (opacity) is another evident effect. While the color changes are less significant at 50 kGy, PS and PET fragments may still undergo slight oxidation, leading to a yellowish tint, and transparent fibers could become mildly opaque.

Since the results can vary widely depending on the conditions indicated, there is currently no acknowledged percentage for the disintegration of MPs exposed to gamma‐rays. It should be mentioned that research in this field is still in its early phases and that exact data on how well gamma‐rays break down MPs are currently accessible. Plastics can decompose with the aid of environmental factors including ultraviolet (UV) light that encourages oxidative decomposition.^[^ ^26^, ^27^, ^30^ ^]^

## 3 Conclusion

Thanks to gamma‐ray irradiation, the degradation capability of MPs was analyzed in this study, achieving a maximum degradation percentage of roughly 70%. In terms of MP fibers and fragments degradation, the results show that the two MPs forms exhibit the same process, with a maximum degradation rate of 35% for both MPs shapes. The purpose of our hypothesis was to assess the level of MPs degradation at various gamma irradiation dosages. Our goals were to examine the impact of gamma irradiation on the breakdown of microplastics in secondary sludge and to examine the mechanical mechanisms involved in this process. Results here obtained reveal the interest in the possibility of using γ‐irradiation to treat sludge before introducing it into the environment as a way to help in the urgent issue of stopping MP pollution. Ideally, ^60^Co seems the best choice for eliminating MPs because of its high energy, stability, and effectiveness in producing free radicals. However, a number of variables, like the type of MPs, the available funds, and the infrastructure, will influence the final decision. The approach for the sustainable treatment of plastic trash found in sewage sludge is developed in part by this study. Further investigations are necessary, in particular, to determine the interactions of sludge‐MPs during the irradiation treatment and their effect on the efficacy. It is expected that future works can improve the irradiation performance on MP removal by optimizing dosages, and exposure durations, and combining it with other treatments. This study offers alternative solutions for environmental health, assisting in the reduction of MPs in sludge and preventing their discharge into wetlands and agricultural soils. Additionally, it stops the propagation of other contaminants, such as heavy metals and hazardous chemicals, that are transported by MPs. In engineering, gamma irradiation may be employed as a sophisticated or supplementary method of treating sludge and wastewater. The outcome would be a higher‐quality sludge that could be used again in farming. Gamma irradiation may provide a less disruptive and more efficient solution in terms of reagent use as compared to mechanical or chemical techniques.

## 4 Experimental Section

γ‐irradiation was used in the study as a treatment method for MPs present in secondary sewage sludge. In the experiment, MPs of different sizes and shapes were exposed to controlled gamma radiation conditions to determine their degradation rates.

### Recovery of MPs

The MPs investigated in this study were collected from secondary sludge samples from an urban WWTP in northwest Spain that serves 8 4000 population equivalents and has an average daily flow rate of 2 3786 m^3^. The mixture of MPs used in the experiments was obtained from thickened sludge samples taken in the dissolved air flotation (DAF) that thickened the secondary sludge. To prevent MP contamination, all the distilled water and reagents (ZnCl_2_ solution and Fenton reagent) were first bifiltered through a glass microfibre filter (0.7 µm pore size, Whatman, Clifton, NJ, USA). A wet‐weight sewage sludge sample (100 g) was oxidized with a 50% H_2_O_2_ (VWR Chemicals, Briare, France) solution for 24 h at room temperature. Subsequently, 40 mL of Fenton reagent was utilized to guarantee the oxidation of all organic contaminants for a whole day at room temperature. Then, the remaining solids were recovered by using a stainless‐steel sieve module (CISA Sieving Technologies, Barcelona, Spain) with overlapping sizes of 500, 250, 100, and 20 µm. The solid retained on each sieve was washed away with bifiltered distilled water and collected into beakers. Using a ZnCl_2_ solution (d = 1.5 g mL^−1^, 97% purity, VWR Chemicals, Briare, France), MPs were separated from the inorganic impurities by flotation. The supernatant was then vacuum‐filtered using a glass microfibre filter (0.7µm pore, Whatman, Clifton, NJ, USA). This methodology was followed for both recovering MPs for irradiation experiments and MP quantification.

### Microplastic Analysis

A high‐resolution color digital camera (Leica DFC310FX, Leica Microsystems CMS GmbH, Wetzlar, Germany) was linked to a semi‐automatic stereomicroscope (Leica M205FA, Leica Microsystems CMS GmbH, Wetzlar, Germany) to process photographs with a maximum resolution of 1,392 × 1,040 pixels (1.4 Megapixel CCD, objective 1×, zoom 34×, and work distance of 61.5 mm). This allowed for the examination of filters with MPs. Based on size and form, the abundance and total quantity of MPs in each sample were quantified. The fiber and fragment sizes were also computed using the ImageJ program (Confocal UniOvi ImageJ, LAS V4.0 Leica Application Suite, Version 4.0.0), which was made available by the Photon Microscopy and Image Processing Unit of the University of Oviedo.

The MPs were characterized chemically and physically using scanning electron microscopy (SEM). SEM images of the MPs were obtained for this investigation using a JEOL 6610 LV SEM (JEOL Ltd., Tokyo, Japan) equipped with a secondary electron detector and microanalysis.

Using a µ‐FTIR (Perkin Elmer Spotlight 200i FTIR spectrophotometer, Springfield, IL, USA), the chemical composition of the MPs was determined (Figure S1, Supporting Information). The IR microscopy system used transmission as the analytical method. The functional groups and chemical composition of MPs were contrasted with a database spectrum of various compounds found in the apparatus itself. The chemical composition of MPs had been analyzed by the Universidad Autónoma de Madrid (UAM) Molecular Spectroscopy Unit using a Perkin Elmer Spotlight 200i FTIR spectrophotometer (µ‐FTIR analysis); the analysis was carried out by transmission. A spectral database containing over 36,000 spectra of different compounds was used for the identification of different polymers. The analysis of the MPs was carried out under the following measurement parameters: number of scans (30), resolution (16 cm^−1^), spectral range (4000–550 cm^−1^), and aperture of the infrared beam (20 × 100 microns for MP‐fibers and 50 × 50 microns for MP‐fragments). For the Spotlight 200i measurement equipment from Perkin Elmer, the Spectrum software was known as the spectral database. Based on the Sadtler algorithm, it searched through over 36,000 spectra.

### Quality Assurance and Quality control (QA/QC)

Quality assurance and control (QA/QC) was carried out, according to several authors, from MP sampling to quantification.^[^ ^31^, ^32^, ^33^ ^]^ MPs might be released during sample preparation and sampling when plastic bottles and Falcon tubes were utilized. Control trials were thus conducted in duplicate. For each plastic piece, the average MP concentration released was 3.33 ± 0.58 and 3.67 ± 1.15 MPs L^−1^, respectively. This contribution was consistent with other research, accounting for less than 1% of the total MP concentration employed in each experiment.^[^ ^33^, ^34^ ^]^

### Physicochemical Parameters

Total suspended solids (TSS), pH, and humidity were the physicochemical properties of the sludge that are assessed (Table S1, Supporting Information). A simple desktop pH meter (ORP sensION+ PH3, HACH) with a refillable double junction pH electrode (+CAT 5014T, HACH) with an Ag+ barrier was used to test pH (in triplicate). In addition, the size of sludge flocs was measured using a semi‐automatic stereomicroscope (Leica M205FA) equipped with a high‐resolution digital color camera (Leica DFC310FX; 1.4 Mpixel, CCD), using the ImageJ (Confocal UniOvi ImageJ) software. Table 1 represents the continuous 6 months of sample collection from the WWTP and the total suspended solids (TSS) (in g per liters), pH, and moisture content present in the samples. These physicochemical analyses and studies for their natural abundance were the basic inputs for further analysis and treatments.

### Gamma‐Ray Experiment

The γ‐irradiation of the MPs samples in the filters was carried out in a wet well panoramic gamma irradiator using a ^60^Co source at a dose rate of 0.76 Gy min^−1^. Glass fiber filters (see sub head “Quality assurance and Quality control (QA/QC)” in Experimental Section) containing MPs were cut in half. One part was poured into a closed 40 mL Falcon tube containing 20 mL of deionized water and kept as a reference. The other half was placed in a similar container with the same amount of water and submitted to γ‐irradiation. Both samples and their references were analyzed after the same amount of time to reach the wanted total gamma‐ray dose. All γ‐irradiation experiments were performed at the Laboratory of LENA (Laboratorio Energia Nucleare Applicata), which is an “Interdepartmental Service Centre” of the University of Pavia (Italy) that manages a nuclear research reactor.

### MPs Degradation

To calculate the rate of MP degradation after gamma irradiation, the mass loss and the amount of MPs on the filters were input into the following formula:

(1) $$\mathrm{Degradation}\;\mathrm{rate}\;\left(\%\right)=\frac{M_{i}-M_{f}}{M_{i}}\times 100$$

where, M_i_ is the initial mass of MPs before irradiation and M_f_ is the final mass of MPs after irradiation.

### Ethics Approval Statement

All authors agreed to publish, and this research article was never published anywhere else.

### Clinical Trial Registration

There are no clinical trials performed in this research article.

## Conflict of Interest

The authors declare no conflict of interest.

## Author Contributions

D.V. did conceptualization, methodology, wrote the original draft, reviewed and performed editing. C.C. did data curation, formal analysis, visualization, and wrote the original draft. D.D. did funding acquisition, reviewed and performed editing. A.L. and M.D. did supervision, review, and editing.

## Supporting information



Supporting Information

## Data Availability

The data that support the findings of this study are available from the corresponding author upon reasonable request.
