# Supplementary material for: A Ray of Hope: Gamma Radiation for Microplastic Remediation
Source: Glob Chall. 2025 May 7;9(6):2500117. doi: 10.1002/gch2.202500117 (PMC12151798; doi:10.1002/gch2.202500117)
Supplement: Supplementary file 1 — Supporting Information [file GCH2-9-2500117-s001.docx]

**Supporting Information**

A Ray of Hope: Gamma Radiation for Microplastic Remediation

Dhanalakshmi Vadivel,^1*^ Claudio Casella,^2*^ Adriana Laca,^2^ Mario Díaz,^2^ and Daniele Dondi^1^

^1^Department of Chemistry, University of Pavia, Viale Taramelli 12, 27100 Pavia, Italy

^2^Department of Chemical and Environmental Engineering, University of Oviedo, C/ Julián Clavería s/n, 33006, Oviedo, Spain.

*Corresponding author: [dhanalakshmi.vadivel@unipv.it](mailto:dhanalakshmi.vadivel@unipv.it)


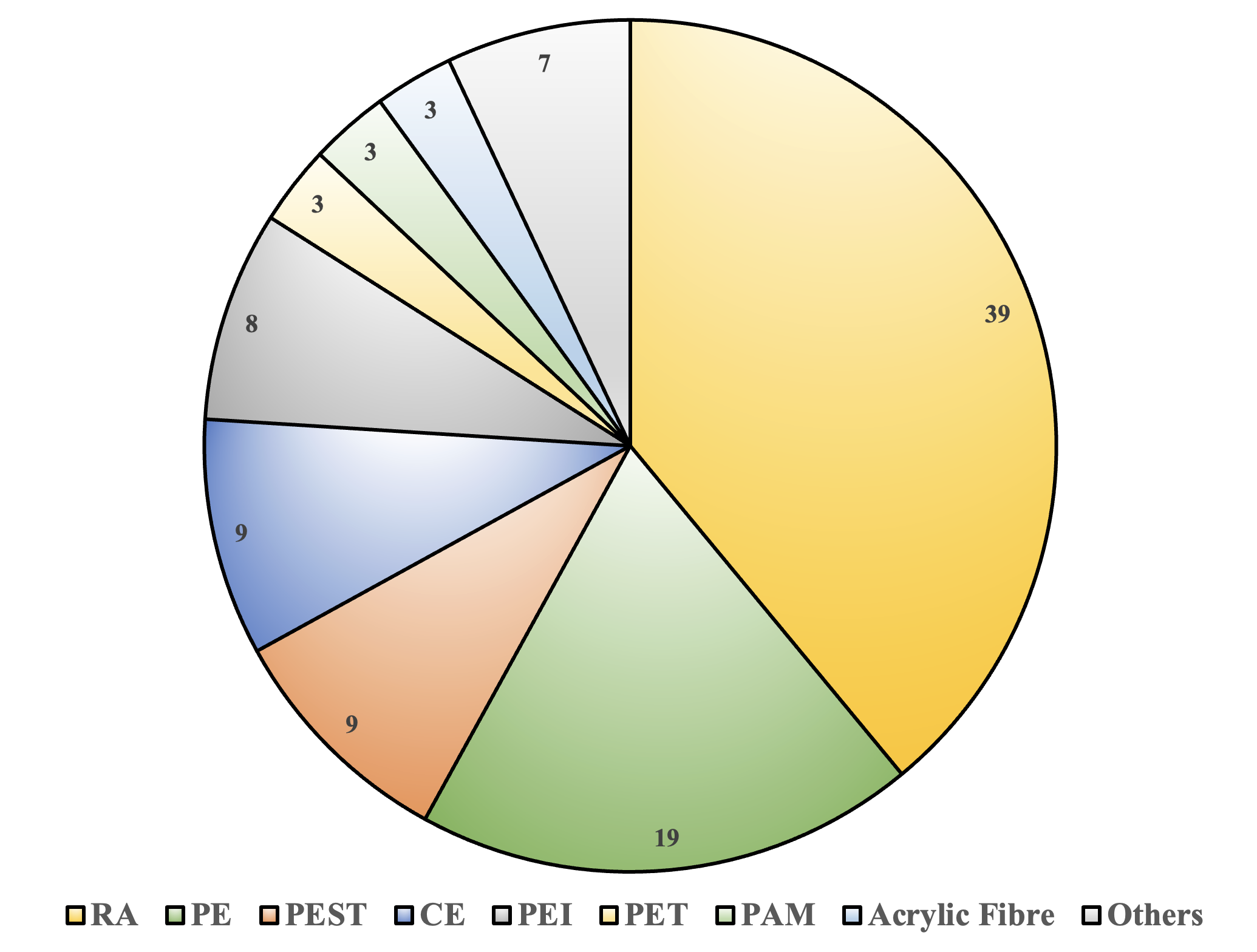


**Figure S1. %** Chemical composition of MPs analysed in secondary sewage sludges

**Table S1.** Physicochemical parameters of the secondary sewage sludge from the WWTP analyzed

| **Month (2023 - 2024)** | **TSS (g/L)** | **pH** | **Moisture (%)** |
| --- | --- | --- | --- |
| November 2023 | 32.6 ± 2.6 | 7.1 ± 0.2 | 93.5 ± 2.3 |
| December 2023 | 33.6 ± 1,9 | 7.4 ± 0.4 | 94.4 ± 1.7 |
| January 2024 | 33.7 ± 2.2 | 7.2 ± 0.1 | 93.4 ± 2.1 |
| February 2024 | 34.2 ± 2.9 | 7.1 ± 0.2 | 93.9 ± 2.4 |
| March 2024 | 33.9 ± 1.6 | 7.2 ± 0.3 | 94.1 ± 1.6 |
| April 2024 | 34.8 ± 2.1 | 7.4 ± 0.1 | 94.5 ± 1.8 |
